# A year in transition: a qualitative study examining the trajectory of first year residents’ well-being

**DOI:** 10.1186/1472-6920-13-96

**Published:** 2013-07-10

**Authors:** Christopher Hurst, Deborah Kahan, Mariela Ruetalo, Susan Edwards

**Affiliations:** 1 Office of Resident Wellness, Postgraduate Medical Education, University of Toronto, 500 University Avenue, Suite 602, Toronto, Ontario M5G 1V7, Canada; 2 McMaster University, 1280 Main Street West, Hamilton, ON L8S 4L8, Canada

## Abstract

**Background:**

It is generally understood that trainees experience periods of heightened stress during first year residency, yet there is little information on variations in stress and well-being over the transition period or those factors that contribute to these variations. This qualitative study explored the trajectory of well-being described by first year residents in the context of challenges, supports and adaptations over time.

**Methods:**

In-depth interviews were conducted face-to-face with 17 first year residents at the University of Toronto. Participants drew a graph of their well-being over the course of their first year and described critical periods of challenge and adaptation. Interviews were audio-taped and transcribed. Results were organized into a thematic analysis using NVivo software.

**Results:**

Residents described a pattern of well-being that varied in accordance with changes in rotations. Well-being increased when residents perceived high levels of team support, felt competent and experienced valued learning opportunities. Well-being decreased with low team support, heavy work demands, few learning opportunities and poor orientations. Anxiety and excitement in the beginning of the year gave way to heightened confidence but increased fatigue and apathy towards the year’s end. Residents used a number of cognitive, behavioural and self-care strategies to cope with transitional challenges.

**Conclusions:**

Residents experienced a pattern of highly fluctuating well-being that coincided with changes in rotations. Residents’ well-being varied according to levels of supervisor and colleague support, learning opportunities, and work demands. Residents’ well-being may be improved by program interventions that facilitate better team and supervisory supports, maintain optimal service to learning ratios, establish effective fatigue and risk management systems, offer wellness support services and integrate skills based resiliency training into the curriculum.

## Background

Numerous studies attest to the challenges experienced by residents during their training. Factors such as long work hours, sleep deprivation, high levels of responsibility, debt and career planning contribute not only to stress, but can also lead to depression and anxiety [1-3]. Several studies have noted that residents have a higher prevalence of depression and generalized anxiety than the general population and that burnout is common [4-7]. The period from clerkship to first year residency is a particularly stressful time as the individual transitions from the role of learner into the dual role of learner and health care provider. This transition period is accompanied by markedly increased expectations and responsibility. Several studies have found stressors specific to this transition period such as uncertainty of expectations, struggling with transferring knowledge into practice, having significantly increased on-call responsibilities and experiencing the unexpected or sudden death of patients [8-11].

In addition to describing common stressors in residency, a number of studies refer to the use of coping mechanisms by residents across all years and a few studies describe mechanisms used specifically among first year residents [4,12,13]. For example, Paice *et al.* identified five coping strategies in their quantitative study of newly qualified doctors (first year residents) [11]. They labeled these broad categories as: seeks social support, problem-focused, wishful thinking, blamed self, and avoidance. The most reported coping strategy was “seeks social support”, followed by “wishful thinking” which referred to wishing the situation could be changed or that it would go away. Strategies such as blaming oneself and avoidance were seldom used. Satterfield and Becerra categorized first year residents’ cognitive responses to stress as including reframing, suppression, changing expectations, looking to the future and escape fantasies [14].

Recent research has begun to focus on the “critical incidents” along the training path. These significant events in the lives of early residents can be sources of personal growth and sources of distress [14,15]. Ackerman *et al.* (2009) identified critical incident themes to include life balance, creating connections, emotional responses to patients, building confidence, managing expectations and facilitating teamwork [16]. These themes represent high and low points in the first year of residency as well as conflicts. High points clustered around gaining confidence in knowledge and skills and developing connections. Low points were spread throughout the thematic framework.

The process of transforming from student to doctor is not fully defined by static stresses and responses, yet we are aware of only one study that looked at temporal variations in mood and empathy during the first year of residency [17]. While research is starting to focus more on the evolution from learner to physician, we still know little about the variations in well-being throughout the critical period of first year residency.

### Project goal

This qualitative study aimed to explore the trajectory of first year residents’ well-being and the factors that affected this trajectory. We also investigated the coping strategies used by first year residents in response to stressors during this transition period. The rationale for the study was that, by obtaining a clearer idea of the factors that most influence transitioning residents’ well-being, medical educators and counselors will be better able to serve the needs of first year residents through targeted educational and support services innovations.

### Research questions

1. What changes in well-being did residents experience over the course of the year?

2. What factors do residents identify as contributing to variations in their well-being during the transition period?

3. What common coping mechanisms do residents report using during the transition period?

## Methods

This exploratory study used an in-depth, individual interview approach to develop a thematic analysis exploring well-being and coping strategies used during the transition period for first year residents at the University of Toronto. Ethics approval was obtained from the University of Toronto Research Ethics board.

### Sample

We collected a non-probability convenience sample by sending out an email invitation to all first year residents at the University of Toronto who had graduated from a Canadian medical school. We excluded international medical graduates because of issues unique to these residents. A follow-up email was later sent to recruit a sufficient number of participants to meet saturation. Saturation of themes was reached with 17 participants. Co-investigators used an iterative process to determine when thematic saturation had been reached.

### Data collection

One interviewer conducted all 17 face-to-face interviews. Participants signed consent forms, which included permission to display transition graphs. They then completed interviews of 30–60 minutes duration. The interviews were audio-taped (with participants’ consent) and then transcribed. At the start of the interviews, participants were asked to draw a graph of their well-being over the course of the first year of residency. Interview transcriptions were analyzed midway through for emergent themes and then after all 17 interviews had been done, when it was decided that saturation was reached.

### Interview questions

The interviewer first collected demographic information, including program and age. Participants were then asked to answer the following questions:

1. Can you draw a graph of your well-being over your first year of residency and then explain it?

2. Many residents experience difficulties during their first year of residency. What are some of the challenges you have experienced during your transition from medical school to residency?

3. How did you cope with the challenges of residency?

4. How have you changed over the course of the year?

Probing follow-up questions were then asked in order to explore participants’ responses. Probing questions included themes from the literature, such as work relationships, clinical demands and time pressures, social support and personal adaptation techniques.

### Data analysis

Data were interpreted using an exploratory approach for common themes. The interview data were coded using NVivo. The participants’ discussions of their transition graphs were used to generate further narrative, which was included in the thematic analysis. Co-investigators analyzed the early transcripts separately, highlighting phrases and words to develop initial codes. Researchers then came together to compare findings in order to reach consensus on the coding structures and theme development. Once the interview process was complete, the researchers met to condense coding groups into sub-themes. Identification, development, and refinement of themes occurred before and during the written phase.

## Results

### Response demographics

Respondents were all Canadian Medical Graduates in their first year of residency training. 71% (n=12) were females and 29% (n=5) were males. 71% (n=12) of residents were registered in Royal College of Physician and Surgeons programs, and 29% (n=5) were Family Medicine residents. Participants’ ages ranged from 25 to 31, with an average age of 26.7. Although we were interested in looking at the different experiences of residents by program type (Family Medicine, vs. procedural specialties vs. other specialties), the number of participants in each category was too low to find thematic differences.

### Upswings and downswings

**Figure 1:**
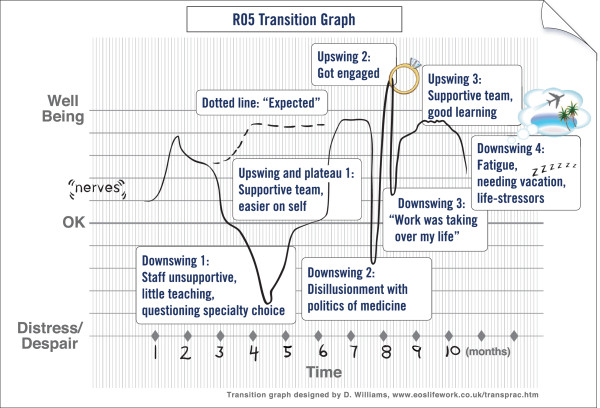
**Graphically modified and annotated example of a transition graph produced by participant R05.** The Y-axis represents well-being, while the X-axis represents time. Residents were free to choose their desired time frame. Most chose months corresponding to changes in rotations to define their X-axis.

The majority of residents started out their training year above or at the baseline level of well-being (See Figure 1). Residents described feeling anxious, and excited about starting a new phase of their professional life. However, they often felt overwhelmed while adjusting to the increased levels of responsibility. This feeling was especially prevalent during residents’ early call shifts.

“I felt like sometimes I was responsible for people’s lives and it was above my ability to take care of them. In retrospect, you always have back-up, we had a fellow in-house and we had a staff to always call. But a lot of times I felt like I just didn't know enough to manage people who I felt were really sick and I felt just very overwhelmed by the responsibility. I couldn't believe it. It was really hard at first.” (R09)

The trajectories recorded in residents’ graphs varied most often with changes in rotations. The rotation system was depicted as a challenge for most residents. With each change in rotation, residents needed to become acquainted with the unfamiliar setting, role expectations, the patients, their electronic records, and the team, while concurrently trying to learn a novel body of knowledge.

“…the first week of any rotation is not even learning about how to treat the patients, it’s about where the charts are kept, who is the best person to speak to, who do you call in this situation, what is your role on the unit? And all of that, you’re learning that on top of learning how to do what you kind of already know how to do. And I think that takes away from… you’re feeling a bit of spaz and not competent and not knowing and asking a lot of questions.” (R08)

While residents had difficulty with the rotation system, they also spoke positively about the finite nature of rotations. Shorter rotations allowed those in a difficult rotation to adopt a ‘wait it out’ attitude and look forward to the end.

Upswings in well-being were often explained as being linked to supportive staff and teams, ample opportunities for learning, increased confidence, lighter rotations and vacations. Residents felt a sense of increasing mastery of the system as they moved through residency and near the end of rotations where they felt more familiar with the people and system they were in.

Residents attributed downswings in well-being to unsupportive staff or team members, long work hours, and call. Other contributing factors identified in the narratives included few opportunities for learning, little interest in the rotation, poor patient outcomes, numerous and complex patients, and fatigue.

While residents frequently reported heightened fatigue towards the end of the year, they also felt more confident and efficient. Interestingly, the majority of residents rated their level of well-being at the end of the year above the baseline.

### Factors affecting upswings and downswings in well-being

#### I. Team support

The degree of team or supervisor support was mentioned most often as a factor influencing resident well-being. When residents experienced a lack of support, their marked well-being on the graph would decrease. Conversely, well-being would increase with strong support. Positive aspects of supervisor and team support included a strong orientation; being highly available and approachable through phone, text, and in person; giving encouraging feedback; making strong efforts at teaching; encouraging residents to ask questions; giving clear expectations; showing interest in the resident as a person; organizing social activities; and providing residents with opportunities to learn through interesting cases or operating room time.

“The hours were maybe even worse, they were really long hours, but because I was being treated with respect and people cared about me, in terms of teaching me how to do things, I loved it.” (R04)

Unsupportive team or supervisor experiences often deeply affected residents. Teams and supervisors could be unsupportive in a number of ways. They gave weak orientations and unclear expectations; were unavailable or unapproachable to residents; discouraged questions; offered vague or negative feedback; did not provide teaching or learning opportunities to residents but expected heavy service work; were disinterested in the resident as a person or did not make efforts to include them; and sometimes harassed and intimidated residents.

“Like, if you didn’t know the blood work at 8:00 in the morning even though it just came out at 7:45, there would be no way of you checking because you are in the operating room the whole time they’ll just tell you that they are disappointed. Or if you’re not suctioning well or you tie something incorrectly, instead of just telling you what you did wrong they’ll take it out on you.” (R04)

#### II. Demands of work environment

Long work hours contributed to expressions of burnout and reduced quality of life. Heavy hours offered residents little time to engage in enriching activities outside of medicine, and often compromised self-care. Of note, most residents preferred long work hours with a supportive supervisor and team to short work hours with unsupportive co-workers.

Call presented a variety of challenges for residents, including a heightened sense of responsibility for patients’ lives, acute clinical situations, negative interactions while on call, and decreased time for self-care. Residents often developed routines for managing the physiological stress of call, such as exercising, and sleeping or eating at specified times. Calls were most overwhelming at the beginning of the year and became easier after repetition.

“The first couple of calls, probably the first five calls, you’re also really tired the next morning because you haven’ t slept… I would walk in the door and I would be bawling… I would just say I feel like I’m literally in charge of people’s lives and I feel like I’m overwhelmed by that. I think 50% of it probably was just fatigue and just the release of coming off of call and the release of all that stress that you’ve built up overnight because you’re so afraid.” (R09)

Fatigue was a common complaint especially towards the end of the year. Residents described themselves as feeling burnt out, and especially tired from the long work hours and call. This affected their well-being and contributed to apathy.

“I’ve heard it’s a common time of the year to get a bit tired. There’s a lot of adrenaline at first, a lot, and yeah, I’m just a bit more tired… I can feel it during my calls, I’m kind of like , oh, yeah, so another consult… I’m just generally a little bit more apathetic about going to work and I’m a bit sick of doing calls.” (R09)

Patient care was also an important work demand. Residents often felt beleaguered by the number and medical complexity of patients. Family Medicine residents spoke about the importance of agenda setting with patients who came to appointments with numerous medical concerns. When patients died or there was another type of negative patient outcome, most residents experienced grief or other forms of distress. Some worried that their patient management was not optimal. Many reflected on the events that took place, often with staff, fellow residents, or family and friends. Reflecting helped residents begin to gain a new perspective about experiences of loss, negative outcomes and their personal feelings of guilt and questions about responsibility for poor outcomes.

“She ended up having like a terrible, terrible complication and dying actually in the end from it… looking back at it and when everyone analyzes, there’s nothing that I could have done to predict or could have done differently to affect any of the outcomes*.*” (R01)

#### III Professional confidence and learning opportunities

Over the course of the year, the transition from anxious uncertainty to a comfortable familiarity with different rotational settings, team and supervisory personnel, clinical protocols and role expectations influenced residents’ appraisals of their levels of stress and well-being. Early on, many residents struggled with concerns that their knowledge or technical skills were not adequate to meet the clinical challenges of rotations. These concerns elicited heightened levels of stress. This was especially pronounced during the early phases of rotations. At times, residents felt that they had just attained a measure of confidence and competence near the end of a rotation and then had to leave.

“When I first got there I didn’t know what a PEEP was or pressure control… like I didn’t know what vent settings were. So by the end for sure you have all the lingo down of the specialty that you’re rotating with. And then it’s kind of sad because you have to leave, like after you kind of feel like you know what you’re doing.” (R03)

Residents referred to periods of increased confidence and lowered stress levels when they “learned the system”. Examples of “learning the system” were: knowing where to send patients for x-rays, what forms to fill out, and where to get help when needed. They also reported higher confidence levels when they worked with familiar teams and knew the expectations.

“And then I had a rotation back … We do three ward rotations, so this was my third time doing it, and I felt very comfortable on the ward. I got to work with the same nurses and the same allied health people. I got to work with my same colleague that I worked with in my first month in July, so everything meshed really well and worked really well, and I find I do a lot better once I know what’s coming at me. So I really enjoy that rotation.” (R07)

Increased levels of confidence also came with opportunities to learn through practice. Not only did exposure to a variety of cases increase confidence, but also the experience of managing them successfully led to a feeling of confidence in being able to handle future cases successfully.

“It was the med-surg ICU and it was really good because it was a lot of learning, a lot of sick, sick patients that you just don’t really come across unless you go to an ICU, like not even on internal medicine are people really that sick. So it was nice to see that, know how those people are managed, get comfortable with the drugs, learn how to intubate, learn how to put in central lines, art lines, those sort of basic skills that you know now that you could do if someone was in trouble.” (R03)

Residents described opportunities for learning, increased familiarity with clinical settings and a growing sense of self-confidence as comforting and leading to better well-being. Conversely, residents experienced decreased well-being on rotations with high work demands and few good learning opportunities. Such rotations reduced residents’ motivation and increased feelings of fatigue. Short staffing was often given as the reason for high levels of scut work and long work hours, especially in surgical specialties.

“… I think the worst thing is to be like an off service person just filling in, just doing the scut work. I think when you sort of take that role that you get the least out of the rotation and it’s also the least satisfying for you, so you learn the least and it’s not a rewarding experience.” (R03)

Please see Table 1 for a summary of the factors that negatively impacted upon resident well-being.

**Table 1:** Take-home point: factors negatively affecting resident well-being

**Factor**	**Description**
Team support	-Weak orientation
	-Difficult to approach
	-Unavailable
	-Gives discouraging feedback
	-Poor effort at teaching
	-Unclear expectations
	-Little interest in resident as a person
	-Few or no social activities
Workplace demands	-Long work hours
	-Call stressors
	-Patient care overwhelming due to complexity or number of patients
	-Negative patient outcomes
Confidence and competency	-Concerns that skills may fall short of rotation expectations
	-Affected by frequently changing rotations
	-Imposter syndrome
Learning opportunities	-Less learning on service heavy rotations
	-Few learning opportunities negatively influenced motivation
	-Short staffing often contributing to scut work

### Coping during upswings and downswings

Residents used cognitive, behavioural, and self-care strategies to cope with the challenges of the transition period.

#### I. Cognitive

Cognitive strategies included self-reflection, reframing, self-talk, not thinking about work and desensitization. Self-reflection was used by residents to work through mistakes and management decisions, and allowed them to broaden their repertoire of options for future clinical situations.

Reframing was used to gain a more balanced perspective on assessment of self and others.

“… I felt like, okay, now we can write prescriptions, now you’re expected to function, you go from medical student to M.D. overnight… [But] the whole point of Residency is to smoothly transition you into that and they don’t expect you to know what you’re doing right off the bat. Whereas, I had very high expectations of myself coming in and I think I put a lot of pressure on myself. So… take a step back, whatever your expectation is, cut it by 50%, and if you’re functioning at 50%, you’re probably at the level that they expect you to be at.” (R12)

Self-talk could be adaptive or maladaptive. Adaptive self-talk tended to be motivational and encouraging. Sometimes self-talk was more likely to be judgmental and critical.

“If I had a bad evaluation, that would be a huge source of stress. It's never happened to me, I can imagine because I evaluate myself very tough sometimes, most of the time.” (R09)

Residents gave examples of consciously not thinking about work and entering into distracting personal activities in order to separate from the day’s events. Some residents described incidents where they became desensitized to patient death and suffering due to the frequency of these occurrences compounded by stress, time pressures and workload. Residents also described becoming less sensitive to negative work interactions and negative feedback.

“But if you have a million consults at night on a service… if you go up to do the consult finally and they're like oh, we're withdrawing care, he's palliative, he's dying, you're kind of like, oh, thank god, awesome… it's a relief because it means sometimes it's just a little bit less work in that moment.” (R09)

#### II. Behavioural

Assertiveness and boundary setting were noteworthy behavioural strategies residents used to cope. Residents demonstrated assertiveness by asking for help, giving feedback, taking initiative, standing up for clinical decisions, advocating for self-care and triaging clinical problems. Boundary setting occurred with patients and at work. Residents created agendas with patients and asserted appropriate appointment lengths. They left work at a designated time, fought for regulated work hours, and took their allotted lieu days and vacations.

#### III. Social

Talking to friends, family and partners about work-related stressors helped residents cope with their situation. Friends who were also in the medical field were particularly useful for understanding the stressors unique to medical residency. Friends and family outside of medicine were more helpful for support and respite from the realm of medicine. Family and partners helped with instrumental supports, including food purchase and preparation, transportation, cleaning and financial errands.

“There were so many things, like little things that you need to do and I wouldn’t do it. And my boyfriend had to do it for me, whether that be depositing things in the bank or a lot of financial things that I just didn’t care about… I remember being like oh, everything is just too overwhelming. I can’t deal with it, like once you’re home, oh my god, change the sheets, but then you’ve got to take the sheets off and then oh, oh my god, let [my boyfriend] do it.” (R14)

#### IV. Self-care

Self-care strategies included exercise, sleep, healthy food preparation, hobbies and meditation or religion. A number of residents used exercise as a time for reflection. Residents also noted that exercise had positive effects on their stress level and mood.

“The way I generally cope with things is I go for runs after call and I just work things through in my brain. I basically re-play everything in slow motion and see what I can do and what I could have done better or what I couldn’t have done anyway.” (R02)

With regards to maladaptive coping strategies, a minority of residents described using alcohol to alleviate stress, but none endorsed the use of illicit drugs or prescription medications as a means of coping. More common was the use of food as a means of reward or stress relief.

## Discussion

The themes identified in our study suggest that from the trainee perspective, the clinical environment plays a significant role in the health and successful adaptation experiences of first year residents. The Conservation of Resources Theory (COR), a leading psychological model for understanding stress and burnout, maps well onto our interpretations of the resident experience. COR theory posits that stress levels and well-being are affected by changes in resource levels. In the COR model, resources may be valued objects (e.g., good housing close to hospitals and transportation), conditions (e.g., positive team support, academic standing) personal skills and traits (e.g., affability, self-confidence) and energy (e.g., physical and cognitive alertness, appropriate clinical knowledge) [18,19]. Resource loss in work settings is associated with increased stress and reductions in well-being. Resource gains are viewed as having a positive effect on well-being and contributing to greater work engagement. Burnout is understood to be the result of a slow, long term depletion of resources without replenishing resource gains [20].

Much like the upswings and downswings depicted in the residents’ transition graphs, COR describes loss or gain spirals where sequences of environmental and personal factors travel together to produce accumulating losses or gains in resources and well-being [21]. From a COR perspective, resources such as effective program orientations, positive supervisory relationships, learning opportunities, and scheduled vacation time, travel together with growth in clinical knowledge and rising self-confidence to produce upswings in well-being. In contrast, high work demands, low organizational supports, problematic supervisory relationships, diminished learning opportunities and reduced time for self-care activities travel together with increasing doubts about self-efficacy and lower work engagement leading to reductions in well-being and personal resource reservoirs. Through the lens of COR theory, resident well-being is seen as closely linked to changes in resource levels associated with the learning environment and not solely the outcome of individual self-care activities or personal characteristics.

Our study found that residents experienced a pattern of highly fluctuating well-being that coincided with changes in rotations and resources. Team support was a critical factor in these variations. Although examples of team support involved fellow-residents, nursing and other inter-professional team members, the most integral relationship was that of resident and supervisor. Effective supervisors act as supportive mentors, engaged clinical teachers and exemplary role models. When faculty members fail to provide effective and engaged supervision, residents are often deeply affected [22-24]. Residents described the need for supervisors who are available and approachable, give constructive feedback, make strong efforts at teaching, and give clear expectations. Faculty development programs teaching a better understanding of learning theory and effective supervisory skills would likely improve the learning trajectory of trainees and assist in promoting the well-being of trainees [22].

Work demands, including workload, work hours and call responsibilities, were a major source of stress for residents. Therefore it is not surprising that residents tended to describe decreased well-being on work-heavy rotations. Work hour regulation is often suggested as a means of addressing the impact of high work demands on residents’ well-being and patient safety. There is some evidence to suggest that work hour regulation may improve the quality of residents’ lives. However, the issue of optimal resident duty hours and sufficient learning opportunities, especially in the procedural disciplines, requires further research [25-27].

Whether work hour regulations are implemented or not, growing evidence suggests that addressing fatigue in the work place is a complex issue that requires more than a reduction in work hours to achieve effective results. Among high-risk industries ( e.g., aviation, oil and gas), attempts to understand and address the complexities of physiological and cognitive fatigue have led to the implementation of Fatigue Risk Management Systems (FRMS) [28-32]. These systems establish on-going organizational structures to support alertness, monitor fatigue levels and actively manage excessive risks of fatigue. FRMS may be one of the next best steps toward addressing the issues of fatigue and burnout in healthcare organizations [27].

Residents referred to the use of a variety of coping mechanisms to manage the stress of residency. Cognitive coping mechanisms included self-reflection, cognitive reframing, self-talk, revising self-expectations, and desensitization. Satterfield and Becerra found similar mechanisms in their 2010 study [14]. Residents also described considerable use of exercise and social interactions with friends and family to ameliorate the stress effects of training. Several residents referred to their exercise routines as a time for reflection that allowed for a positive shift in mood. These finding are similar to Markwell and Wainer’s (2009) survey of junior physicians, where residents referred frequently to the use of exercise and relationships with family and friends as key well-being supports [3].

Although residents described the use of active coping strategies and healthy self-care habits, they experienced considerable variation in well-being. COR theory suggests that when sufficient resource losses occur, active coping and work-engagement decline as part of the downward spiral. At such vulnerable times, wellness programs and services that offer additional resources to residents may be especially helpful in promoting upward spirals of resource gain and well-being.

Levey (2001) and Lefevbre (2012) have proposed the implementation of resident wellness programs as a promising avenue to target residents’ stress and decreased well-being [1,5]. A number of programs offering resident supports and preventative approaches to wellbeing have been documented in the literature [33-36]. The increasing presence of such programs within medical education is encouraging. Future directions in resident wellness programs will ideally reflect greater breadth of support services for residents and the inclusion of a skills-based preventative approach to well-being within the formal academic curriculum [37].

The study has several limitations. Interviews with residents were carried out later in the academic year and represent a retrospective account of the training experience. Interviewing residents several times throughout the year may have offered a more ‘experience near‘ description of residents’ challenges and adaptations. Although a number of the themes related to coping are consistent with previous findings in the literature, the rotational factors identified by residents as affecting their well-being may not be transferable to other institutions.

### Future research

The themes developed in this study suggest several future research directions. Faculty and residents may benefit from research into common interactional patterns within the supervisory process and their effects on clinical performance and well-being outcomes. An exploration of how physicians learn to sustain well-being in demanding environments might offer further information for curricular innovations. Finally, it would be interesting to extend this research by using COR theory to explore the well-being trajectory of physicians during their first year of independent practice.

## Conclusions

First year residents recount substantial variations in their well-being which they link to specific factors in their frequently changing work and training environments. Our thematic findings suggest that postgraduate medical education may improve residents’ well-being by focusing on strategies to increase the quality of supervisory supports, promote optimal service to learning ratios, establish organizational approaches to fatigue and champion resident support services and curricular interventions.

When the training environment is viewed from a COR perspective, improving resident well-being is clearly linked to organizational interventions that foster, support and enrich resources at the individual, team, program, curriculum and institutional levels. Resident well-being is connected to multiple environmental factors and so requires a plurality of interventions from committed and supportive leadership at all levels.

## Competing interests

The authors declare that they have no competing interests.

## Authors’ contributions

CH conceptualized this study, led the design of the research methods, helped train the interviewer (DK), contributed to the analysis of results, and co-drafted the manuscript. DK contributed to the study design and to the analysis of results, conducted the interviews, and co-drafted the manuscript. MR contributed to the study design, the analysis of results, trained the authors on the use of NVivo, and edited the manuscript. SE contributed to the study design, to the analysis of results, and edited the manuscript. All authors read and appproved the final manuscript.

## Pre-publication history

The pre-publication history for this paper can be accessed here:

http://www.biomedcentral.com/1472-6920/13/96/prepub
